# Exploring the Mutational Landscape of Isolated Congenital Heart Defects: An Exome Sequencing Study Using Cardiac DNA

**DOI:** 10.3390/genes13071214

**Published:** 2022-07-07

**Authors:** Ilse Meerschaut, Wouter Steyaert, Thierry Bové, Katrien François, Thomas Martens, Katya De Groote, Hans De Wilde, Laura Muiño Mosquera, Joseph Panzer, Kristof Vandekerckhove, Lara Moons, Petra Vermassen, Sofie Symoens, Paul J. Coucke, Daniël De Wolf, Bert Callewaert

**Affiliations:** 1 Center for Medical Genetics, Ghent University Hospital, 9000 Ghent, Belgium; ilse.meerschaut@ugent.be (I.M.); wouter.steyaert@radboudumc.nl (W.S.); laura.muinomosquera@uzgent.be (L.M.M.); lara.moons@ugent.be (L.M.); petra.vermassen@ugent.be (P.V.); sofie.symoens@ugent.be (S.S.); paul.coucke@ugent.be (P.J.C.); 2 Department of Pediatric Cardiology, Ghent University Hospital, 9000 Ghent, Belgium; katya.degroote@uzgent.be (K.D.G.); hans.dewilde@uzgent.be (H.D.W.); joseph.panzer@uzgent.be (J.P.); kristof.vandekerckhove@uzgent.be (K.V.); daniel.dewolf@uzgent.be (D.D.W.); 3 Department of Human Genetics, Radboud Institute for Molecular Life Sciences, Radboud University Medical Center, 6525 GA Nijmegen, The Netherlands; 4 Department of Cardiac Surgery, Ghent University Hospital, 9000 Ghent, Belgium; thierry.bove@uzgent.be (T.B.); katrien.francois@uzgent.be (K.F.); thomas.martens@uzgent.be (T.M.); 5 Department of Pediatric Cardiology, Brussels University Hospital, 1090 Brussels, Belgium

**Keywords:** congenital heart defects, exome sequencing, somatic variation, oligogenic inheritance, polygenic inheritance, transmission disequilibrium testing, association testing

## Abstract

Congenital heart defects (CHD) are the most common congenital anomalies in liveborn children. In contrast to syndromic CHD (SCHD), the genetic basis of isolated CHD (ICHD) is complex, and the underlying pathogenic mechanisms appear intricate and are incompletely understood. Next to rare Mendelian conditions, somatic mosaicism or a complex multifactorial genetic architecture are assumed for most ICHD. We performed exome sequencing (ES) in 73 parent–offspring ICHD trios using proband DNA extracted from cardiac tissue. We identified six germline de novo variants and 625 germline rare inherited variants with ‘damaging’ in silico predictions in cardiac-relevant genes expressed in the developing human heart. There were no CHD-relevant somatic variants. Transmission disequilibrium testing (TDT) and association testing (AT) yielded no statistically significant results, except for the AT of missense variants in cilia genes. Somatic mutations are not a common cause of ICHD. Rare de novo and inherited protein-damaging variants may contribute to ICHD, possibly as part of an oligogenic or polygenic disease model. TDT and AT failed to provide informative results, likely due to the lack of power, but provided a framework for future studies in larger cohorts. Overall, the diagnostic value of ES on cardiac tissue is limited in individual ICHD cases.

## 1. Introduction

Congenital heart defects (CHD) are defects in the structure of the heart or the great vessels that develop during early fetal life. They form a major cause of spontaneous abortion, stillbirth, and termination of pregnancy, and affect around nine per 1000 live births [1,2]. The clinical spectrum of CHD is heterogeneous, ranging from simple defects to complex multi-structural deformities. The majority of CHD in liveborns appear isolated (ICHD), with associated extracardiac manifestations observed in approximately only 25% of cases [3]. In contrast to the tremendous progress in deciphering syndromic CHD (SCHD) cases, the genetics underlying ICHD remain largely unresolved. Nonetheless, better knowledge of the molecular mechanisms in ICHD is important for patients and their relatives, as it would allow for more precise counseling on disease prognosis and familial recurrence risk, and might advance treatment and prevention strategies. ICHD can present as simple Mendelian traits, but this one gene–one disease paradigm is insufficient to explain most ICHD cases. A distinct complex genetic architecture for ICHD is suggested by observations from twin studies, the recurrence risks in siblings and offspring of CHD patients, as well as the intra- and interfamilial phenotypic variability. This architecture for ICHD ranges from oligogenic to polygenic [4,5,6]. Somatic coding variants have, in addition, been suggested as contributing to the missing heritability of ICHD. Some studies illustrated somatic variants in the cardiogenic transcription factors *NKX2-5* and *TBX5* in formalin-fixed CHD hearts [7,8,9]; however, these findings could not be consistently confirmed in subsequent studies using freshly frozen cardiac tissue. The debate on the role of somatic variants, either as a causal or contributing factor to CHD, has still not been settled [10,11,12,13,14,15,16,17,18,19,20].

We performed an exome sequencing (ES) study in parent–offspring ICHD trios using proband DNA extracted from freshly frozen cardiac tissue and parental leucocyte DNA, aiming to unravel the molecular basis of ICHD. First, we evaluated somatic variants and rare de novo or inherited germline variants, in relation to the ICHD phenotype. We, therefore, focused on rare variants with damaging in silico predictions, occurring in cardiac-relevant genes (e.g., cardiogenic transcription factor genes, genes from gene regulatory pathways involved in heart development, cardiac structural genes, and known CHD genes) expressed in human embryonic cardiac tissue. A further aim was to identify the genetic linkages between ICHD and single genes or gene panels. Therefore, we evaluated the distortion of the transmission of alleles from heterozygous parents to affected offspring using transmission disequilibrium testing (TDT) [21], and searched for associations between ICHD and variants at the gene or gene panel level by performing association testing (AT).

## 2. Materials and Methods

### 2.1. Study Cohort

Patients were recruited from the departments of pediatric cardiology and medical genetics of Ghent University Hospital. Patients with ICHD were eligible for inclusion in the study if a preceding copy number variation (CNV) analysis was normal or not explanatory for the CHD, and if a cardiosurgical intervention was planned, enabling the collection of cardiac tissue. Patients with SCHD or a positive familial history of CHD in a first- or second-degree relative were excluded. The availability of blood samples of both parents was required for parent–offspring trio analysis.

For AT, we used ES data of a control group composed of patients with a suspected Mendelian genetic disease without cardiac involvement. Genetically related individuals were excluded based on the construction of a genetic relationship matrix with the smartpca program from the EIGENSOFT software package version 7.2.1 [22]. All samples having a genetic relationship value (with any other sample in the group) larger than the smallest relationship value between a sample with itself were excluded.

This study was approved by the Ethics Committee of Ghent University Hospital (EC 2014/0854 and EC 2019/1621). Written informed consent was obtained for all ICHD probands.

### 2.2. DNA Extraction

Proband DNA was extracted from cardiac tissue obtained from the right atrium or the affected cardiac region itself (e.g., in aortic coarctation), which was freshly frozen in RNA*later* Stabilization Solution (Thermo Fisher Scientific, Waltham, MA, USA). For preparation, cardiac samples were lysed at 36 °C for 2.5 h using a lysis buffer (0.1 M NaCl, 40 mM Tris HCl pH 7.0, and 20 mM EDTA pH 8.0), a same volume of equal amounts of phenol, chloroform, and isoamylalcohol was added, and the samples were incubated overnight at 4 °C with gentle shaking. After 2 steps of centrifugation, the lower phase was removed and Chloroform was added overnight, 150 μL of 4 M NaCl and 2 mL of ethanol were added to 1 mL of the DNA-containing upper phase, and the mixture was incubated overnight at 4 °C. The DNA-containing phase was then brought into progressively higher concentrations of ethanol (70% and 100%) and, after drying, it was dissolved in H_2_O.

The DNA of parents and controls was extracted from leucocytes using standard in-house DNA-extraction protocols.

### 2.3. Exome Sequencing

ES was performed on a HiSeq 3000 platform (Illumina, San Diego, CA, USA). DNA enrichment was performed with the SureSelect Human All Exon V6 (SSV6) enrichment kit (Agilent Technologies, Santa Clara, CA, USA). Because the amounts of available proband cardiac DNA were limited (due to the small sizes of the tissue samples), an adjusted 200 ng protocol was used for ES in the case trios. Control samples were sequenced following the standard in-house protocol using 3 μg of DNA.

All samples passed the in-house quality thresholds, i.e., 80% of all sequenced bases were above Q30 and 90% of Agilent targeted bases had a sequence depth above 20.

### 2.4. Raw Data Processing

Sequencing reads were aligned to the human reference sequence GRCh38 with the Burrows–Wheeler Aligner tool BWA-MEM version 0.7.17 [23]. Duplicate reads were marked with Picard tools version 2.18.20 (http://broadinstitute.github.io/picard/) (accessed on 11 June 2022). The Genome analysis toolkit GATK version 3.8 HaplotypeCaller [24,25] was used in the genomic variant call format (GVCF) mode, resulting in per-sample genotype likelihoods. All GVCFs were jointly genotyped using GATK version 4.0.4.0 to end in one variant call format (VCF) file that contained all genotypes for all samples. Variant quality score recalibration (VQSR) was applied with GATK version 4.0.4.0 to the full variant callset. Variants that were not in the top-quality tranche (i.e., sensitivity threshold of 99.9%) after VQSR were excluded for TDT and AT. Finally, all variants were annotated with Ensembl Variant Effect Predictor (VEP92) [26].

### 2.5. GnomAD and CADD

In different steps of the variant analyses, the frequency of variant alleles in gnomAD (gnomAD AF) was used for filtering. For this, we systematically used the global genome population allele frequency from gnomAD version 2.0.1 [27]. Originally, these variants were relative to GRCh37. Since we used GRCh38 in our study, we used the GRCh38 version, which was remapped with CrossMap (ftp://ftp.ensembl.org/pub/data_files/homo_sapiens/GRCh38/variation_genotype/gnomad/r2.1/genomes/) (accessed on 16 March 2019).

This GnomAD version was also used for the extraction of the loss-of-function (LOF) upper bound of the observed/expected (oe) confidence intervals and missense upper bound of the oe confidence intervals.

All CADD annotations were derived from CADD 1.6 [28].

### 2.6. Mosaic Variant Calling

MosaicHunter 1.1 [29] was used to identify de novo somatic variants within the ICHD cases. The MosaicHunter output was annotated with Ensembl Variant Effect Predictor (VEP92). Only variants with a posterior probability of ≥90% were retained. Variants were further filtered on variant allele fraction (VAF) 0.10–0.40, parental background ≤2 alternate alleles at the variant position in the parents, variant recurrence ≤2 in the study cohort, and predicted variant consequence LOF or missense.

LOF variants were the grouping of the following Ensembl consequences: stop gained, stop lost, start lost, frameshift variant, and splice acceptor or splice donor.

Confirmation of the selected potential somatic variants was performed on the MiSeq platform (Illumina, San Diego, CA, USA) with an intended average coverage of 2000–3000× on both proband cardiac DNA and leucocyte (or fibroblast) DNA and parental leucocyte DNA.

Confirmed somatic variants were further evaluated for their potential relevance to ICHD phenotypes based on the expression of the gene in human embryonic tissue (see below), in silico predications (LOF upper bound of the oe confidence interval for LOF variants and missense upper bound of the oe confidence interval and CADD scores for missense variants), and the presence of the genes in CHD-related gene panels (see below). The cutoffs used for the in silico predictors to consider a variant to be ‘damaging’ were a LOF upper bound of the oe confidence interval of <0.35, missense upper bound of the oe confidence interval of <0.35, and CADD ≥ 25.

### 2.7. Trio Analysis

Parent–offspring trio analysis was performed for all included ICHD cases to evaluate the occurrence of high-quality (HQ) de novo and inherited germline variants.

A variant was considered a HQ de novo variant (DNV) if it fulfilled all of the following criteria:

(1) A heterozygous single nucleotide variant (SNV) with GATK’s single sample quality score (QUAL) > 300 or a heterozygous small insertion or deletion (indel) with QUAL > 1000;
(2) Both parents were homozygous for the reference allele with a corresponding genotype quality (GQ) ≥ 30;
(3) The variant was observed only once or twice in our case cohort of 73 ICHD patients.
  Inframe indels were excluded from further analysis because of the high risk of false-positive calls for this variant type. From the remaining HQ DNV, only LOF variants and missense variants with gnomAD AF ≤ 0.1% were retained for further interpretation.

A variant was considered a HQ inherited variant if it fulfilled all of the following criteria:

(1) A SNV with QUAL > 300 or a small indel with QUAL > 1000;
(2) The variant was also present in one or both parents;
(3) The variant was observed only once or twice in our case cohort of 73 ICHD patients.

Again, inframe indels were excluded from the further analysis, and only rare inherited LOF variants and missense variants with gnomAD AF ≤ 0.1% were retained.

The QUAL cut-offs used above were determined based on an in-house validation study and resulted in a sensitivity of 97.3% and specificity of 93.9% for SNVs.

Rare HQ de novo and inherited variants were further evaluated for their potential relevance to ICHD phenotypes, similar to the evaluations performed for the confirmed somatic variants (see above).

### 2.8. Transmission Disequilibrium Testing

TDT was conducted using the FBAT toolkit [30]. The set of parental variants consisted of all HQ rare heterozygous LOF and missense variants in the 146 ICHD parents. LOFs were defined as above in the trio-analysis.

A variant was considered a HQ rare variant if it fulfilled the following criteria:

(1) SNV with QUAL > 300 or a small indel with QUAL > 1000;
(2) The gnomAD AF was ≤ 0.1%.

Inframe indels were excluded from further analysis because of the high risk of false-positive calls. A parental variant was considered to be transmitted to the child if it was present in the variant call set described in Section 2.4.

The genetic positions needed in the analyses were downloaded from the Beagle page (http://bochet.gcc.biostat.washington.edu/beagle/genetic_maps/plink.GRCh38.map.zip) (accessed on 20 September 2018).

TDT analysis was performed at the single-gene level and the gene panel level. The gene panels tested are listed below. For both analyses, only genes being expressed in human cardiac tissue during the embryonic phase (see below) were considered, and LOF variants and missense variants were tested separately. For reasons of statistical power, a test was only conducted when the number of variant alleles of the test was ≥5.

For both the single-gene and gene panel analyses, a correction for multiple hypothesis testing was conducted using the false discovery rate (FDR) approach by Benjamini Hochberg (BH).

### 2.9. Association Testing

AT was performed at the single-gene and gene panel levels. The gene panels tested are listed below. For both analyses, only LOF variants and missense variants with a gnomAD AF of ≤0.1% and a total study cohort frequency (cases and controls combined) of ≤1% occurring in genes being expressed in embryonic human cardiac tissue (see below) were considered. The total study cohort frequency filtering was conducted to rule out recurrent variants resulting from technical in-house artifacts and common variants that were not filtered via gnomAD (as this gnomAD version was a lift-over from GRCh37 to GRCh38). LOF variants and missense variants were tested separately, per gene or gene panel, if the number of variant alleles per full set of cases and controls was ≥5.

Since the population structure can be an important source of bias in AT, principal component (PC) analysis was conducted. This was conducted with the smartpca program from the EIGENSOFT software suite version 7.2.1.

A logistic regression model was applied to the data in which the variant fraction (VF) and PC1 were included as covariates. The linear predictor of the statistical model becomes:

$$\log\frac{p_{i}}{1-p_{i}}=\beta_{0\;}+{\;\mathrm{VF}}_{i}\cdot \beta_{1}+\mathrm{PC}1_{i}\cdot \beta_{2}\;i=1,\;2,\ldots n$$

where ${\;\mathrm{VF}}_{i}=\sum \mathrm{weighted}\;\mathrm{variant}\;\mathrm{allele}\;\mathrm{count}\;\left({\mathrm{VAC}}_{i}\right),$ with the variant weighting schema being as follows: VAC*(CADD/10). In this, CADD refers to the phred-scaled CADD-score.

A likelihood ratio test was used to evaluate whether the regression parameter β_1_ for VF was statistically different from 0 or not. Raw *p*-values were corrected using the BH-approach.

Logistic regression analyses were performed in R using the glm function with the logit link function, and all subsequent likelihood ratio tests were performed with the anova function with test = LRT.

### 2.10. Gene Panels and Gene Expression in Human Heart during Embryonic Development

The gene panels tested in the TDT and the AT included an in-house CHD panel of 471 known or candidate CHD genes, a list of 1639 human transcription factors [31], and fifteen lists of genes annotated to specific CHD-related Gene Ontology (GO) terms (bmp signaling pathway GO:0030509 (152 genes), wnt signaling pathway GO:0016055 (517 genes), notch signaling pathway GO:0007219 (190 genes), transforming growth factor β-receptor signaling pathway GO:0007179 (178 genes), smoothened signaling pathway GO:0007224 (141 genes), nodal signaling pathway GO:0038092 (17 genes), hippo signaling GO:0035329 (38 genes), fibroblast growth factor receptor signaling pathway GO:0008543 (108 genes), vascular endothelial growth factor signaling pathway GO:0038084 (36 genes), ras protein signal transduction GO:0007265 (404 genes), histone modification GO:0016570 (465 genes), chromatin remodeling GO:0006338 (168 genes), cell surface receptor signaling pathway involved in heart development GO:0061311 (30 genes), sarcomere GO:0030017 (204 genes), and cilium GO:005929 (668 genes)). The GO gene lists were downloaded from http://www.geneontology.org (accessed on 1 November 2019) and filtered by organism ‘homo sapiens’ and type ‘protein’ [32]. An overview of the genes contained in each panel is given in Supplementary Table S1.

These panels were further filtered for genes being expressed in human cardiac tissue during the embryonic phase. The expression data of genes during the development of the human heart were obtained from a publicly available RNA-seq time-series dataset covering the development of seven organs, including the heart (http://www.ebi.ac.uk/ —accession number E-MTAB-6814) (accessed on 19 April 2021) [33]. All genes with an expression threshold of two transcripts per million (TPM) in cardiac tissue in at least one developmental stage from ‘four weeks post-conception’ to ‘eight weeks post-conception’ were considered as being expressed in human cardiac tissue during the embryonic phase.

The same gene panels and expression data were used for the further interpretation of the confirmed somatic variants and the HQ rare de novo and inherited variants identified from the trio-analysis.

## 3. Results

### 3.1. Study Cohort

We included 73 patients with genetically unexplained ICHD. For all patients, blood samples, cardiac tissue, and blood samples of both parents were available. The sex ratio was 43 boys to 30 girls. The heart defects involved were the transposition of the great arteries (n = 11) (15.1%), atrial septal defect (n = 11) (15.1%), tetralogy of Fallot (n = 10) (13.7%), ventricular septal defect (n = 10) (13.7%), aortic coarctation (n = 8) (11.0%), functionally univentricular heart (n = 6) (8.2%), left ventricle outflow tract abnormality (n = 6) (8.2%), atrioventricular septal defect (n = 6) (8.2%), double outlet right ventricle (n = 4) (5.5%), and right ventricle outflow tract abnormality (n = 1) (1.4%). An overview of the heart defects in the probands is given in Supplementary Table S2.

The control group for the single-gene and gene panel AT included 1274 unrelated patients.

**Figure 1:**
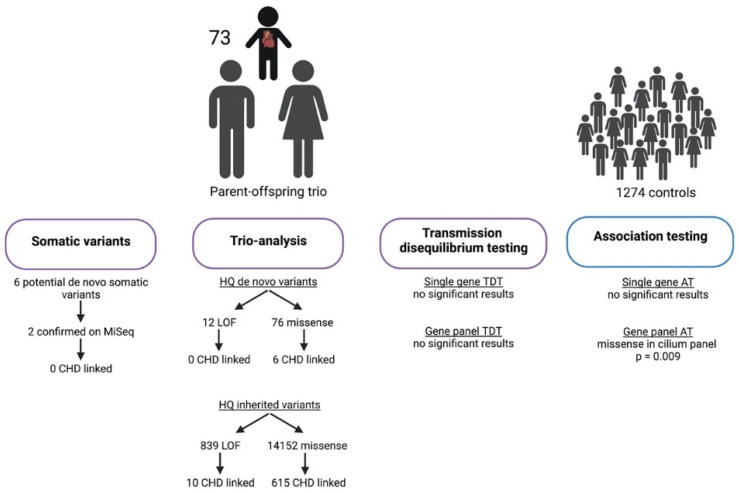
Overview of the study cohort and the results of the study analyses. Figure created with Biorender.com.

An overview of the study cohort and the main results of the study analyses is provided in Figure 1.

### 3.2. Mosaic Variant Calling

The total cohort contained 1550 variants with a posterior probability of ≥90%, according to MosaicHunter. After filtering these variants by VAF, parental background, cohort recurrence, and predicted variant consequence, six potential de novo mosaic variants remained. All of these were missense variants. An overview of these variants and the MiSeq validations is given in Supplementary Table S3.

Two of these six variants (*ABCC2* and *LSP1*) were confirmed mosaic variants in cardiac DNA of the proband (respectively VAF 0.20 and 0.06). Only the variant in *ABCC2* was also confirmed mosaic in leucocyte DNA of the proband. One of the six variants (*RAB11FIP2)* was shown to be a heterozygous variant in both the cardiac and leucocyte DNA of the proband. The *ABCC2* and *RAB11FIP2* variants were also called in the HQ DNV trio analysis (see below). The remaining three variants could not be confirmed in either the cardiac or leucocyte DNA of the proband. None of the validated mosaic variants (*ABCC2* and *LSP1*) were confirmed in the parents, and were thus shown to be true de novo mosaic variants.

The two confirmed mosaic variants both occurred in a gene being expressed during human heart development. Only the variant in *ABCC2* was a rare variant, whilst the *LSP1* variant was a common single-nucleotide polymorphism (SNP) (gnomAD AF 0.32). *ABCC2* had a missense upper bound of oe confidence interval of 1.16, indicating a high occurrence of missense variants in the *ABCC2* gene. Nonetheless, the CADD score of 28.7 suggested that this specific missense variant might have been deleterious. *ABCC2* (MIM 601107) did not occur in any of the studied CHD-related gene panels and a Pubmed search neither revealed a link to heart development or CHD. See Supplementary Tables S3 and S4.

### 3.3. Trio Analysis

#### 3.3.1. High-Quality de Novo Variants

The total cohort contained 355 HQ DNV, ranging from zero to eighteen per sample (median 3, mean 4.86, and standard deviation 4.25). Of these, 179 HQ DNV had a gnomAD AF of ≤0.1%. The latter ranged from zero to thirteen per sample (median 2—mean 2.45—standard deviation 2.25) and included twelve LOF variants and 76 missense variants.

Of the twelve LOF variants, only two variants (*MPP6* and *RAP1GDS1*) occurred in a gene being expressed in human cardiac tissue in the embryonic phase. Only *RAP1GDS1* had a LOF upper bound of the oe confidence interval of <0.35, suggesting sensitivity for haploinsufficiency. *RAP1GDS1* (MIM 179502) has recently been linked to intellectual disability, global developmental delay, and hypotonia [34], but lacks a clear link with heart development or CHD. See Supplementary Table S4.

Of the 76 missense variants, 58 occurred in genes that show expression in human cardiac tissue in the embryonic phase. Of these, nineteen missense variants had a CADD score of ≥25 (*ABCC2*, *ABCF3*, *ALLC* (2×), *AMZ1*, *DENND1C*, *DOCK8*, *DPYSL4*, *FAM83G*, *PLEC*, *RAB11FIP2*, *ROBO1*, *RPS6KA5*, *SAMHD1*, *SDR39U1*, *SLC38A6*, *TBL1XR1*, *WNK4*, and *ZBTB7B*). Two of the missense variants occurred in a gene with a missense upper bound of the oe confidence interval of <0.35, suggesting low tolerance for missense variants in these genes (*RHOA* and *TBL1XR1*). The specific variant in *RHOA* identified here had a CADD score of 22.0, and the specific variant in *TBL1XR1* had a CADD score of 28.8. The two variants in the *ALLC* gene occurred in the same patients and affected subsequent cis-located bases, thus representing one multi-nucleotide variant. The *ABCC2* variant was actually a mosaic variant (see above).

Six of the above genes were present in one or more CHD-related gene panels. *FAM83G* (MIM 615886) was linked to bmp signaling; *RHOA* (MIM 165390) was linked to ras, TGFbeta, and wnt signaling; *ROBO1* (MIM 602430) was linked to notch, ras, and VEGF signaling; *RPS6KA5* (MIM 603607) was linked to histone modification; *TBL1XR1* (MIM 608628) was linked to wnt signaling and histone modification; and *ZBTB7B* (MIM 607646) was a transcription factor and had also been linked to histone modification. An additional Pubmed search suggested an additional link of *RHOA*, *ROBO1*, and *TBL1XR1* to heart development and/or CHD [35,36,37,38,39]. See Supplementary Table S4.

#### 3.3.2. High-Quality Rare Inherited Variants

The number of HQ rare inherited variants in the total cohort was 31134, and ranged from 304 to 978 per sample (median 366, mean 426.49, and standard deviation 153.27). These rare HQ inherited variants included 839 LOF variants and 14,152 missense variants.

Of all 839 LOF variants, 583 variants occurred in a gene that was expressed in human embryonic cardiac tissue. Twenty-seven of these variants occurred in a gene with a LOF upper bound of the oe confidence interval of <0.35, suggesting sensitivity to haploinsufficiency of that gene (*ACIN1*, *AFDN*, *ANKHD1-EIF4EBP3* (2×), *ATF2*, *ATP13A3*, *CELSR3*, *CSMD1*, *EMSY*, *EPHB2*, *FBXO22*, *FNIP2*, *GANAB*, *GNB2*, *HIVEP3*, *LAMC1*, *LRRC8B*, *MSH2*, *POFUT1*, *PRR14L*, *SCAI*, *SCAMP1*, *SLC4A4*, *SOX6*, *THOC1*, and *TSC2* (2×)). The two variants in the *ANKHD1-EIF4EBP3* and *TSC2* gene each affected subsequent cis-located bases in the same patient, and thus formed one multi-nucleotide variant each. Ten of these genes were present in one or more CHD-related gene panels. *ATF2* (MIM 123811) was a transcription factor and a histone-modifying gene, *CELSR3* (MIM 604264) was a wnt signaling gene, *EPHB2* (MIM 600997) occurred in the CHD panel and ras signaling panel, *FBXO22* (MIM 609096) was a sarcomere gene, *HIVEP3* (MIM 606649) was a transcription factor, *LAMC1* (MIM 150290) encoded a basement anchorage laminin expressed in cardiac tissue and was included in the CHD panel, *POFUT1* (MIM 607491) was a notch signaling gene, *SCAI* (MIM 619222) was a ras signaling gene, *SOX6* (MIM 607257) was a transcription factor, and *TSC2* (MIM 191092) was a wnt signaling gene. An additional Pubmed search suggested a potential additional link of *EPHB2*, *SOX6*, and *TSC2* to heart development and/or CHD [40,41,42,43]. See Supplementary Table S4.

Of the 14,152 missense variants, 10,901 variants occurred in a gene that showed expression in human embryonic cardiac tissue. Of these, 2629 missense variants had a CADD score of ≥25 (see Supplementary Table S4). Furthermore, eight missense variants occurred in a gene with a missense upper bound of the oe confidence interval of <0.35, suggesting intolerance for missense variants of that gene (*ACTC1*, *KPNB1*, *NOVA2*, *PSMC1*, *PURA*, *SMC1A*, *STAT1*, and *TUBB*). The specific variant in *ACTC1* had a CADD score of 31 (and was thus also counted in the 2629 missense variants mentioned above), the specific variant in *KPNB1* had a CADD score of 23.6, the specific variant in *NOVA2* had a CADD score of 22.6, the specific variant in *PSMC1* had a CADD score of 22.9, the specific variant in *PURA* had a CADD score of 22.1, the specific variant in *SMC1A* had a CADD score of 16.7, the specific variant in *STAT1* had a CADD score of 23, and the specific variant in *TUBB* had a CADD score of 24.6. The two *GART* variants and the two *SLC45A1* variants each affected subsequent cis-located nucleotides in the same patient, and thus resembled one multi-nucleotide variant each. Of the above variants, 615 occurred in a gene that was present in one or more CHD-related gene panels. An overview of these genes, the CHD gene panels, and the OMIM phenotypes and the results of an additional Pubmed search evaluating potential relations to heart development and/or CHD is given in Supplementary Table S4.

### 3.4. Transmission Disequilibrium Testing

TDT was performed for 2284 genes, thereby testing LOF variants (12 genes) and missense variants (2272 genes) separately. After correction for multiple hypothesis testing, none remained significant at an FDR level of 0.05.

We also performed TDT for 28 CHD-related gene panels, again testing LOF variants (11 panels) and missense variants (17 panels) separately. This did not show any significant results at an FDR level of 0.05 after correction for multiple hypothesis testing.

### 3.5. Association Testing

After the selection of the appropriate variants and selection for minimum variant allele counts, 10,970 genes (896 LOF variant genes and 10,074 missense variant genes) were retained for single-gene AT. After correction for multiple hypothesis testing with the BH procedure, no genes remained significantly associated with the ICHD phenotype at an FDR level of 0.05.

For the gene panel AT, after correction for multiple hypothesis testing with the BH procedure, only the cilium gene panel remained significantly associated with the ICHD phenotype at an FDR level of 0.05 (*p*-value 0.009), with cases harboring fewer missense variants compared with the controls.

## 4. Discussion

We performed a trio ES study on cardiac tissue of 73 sporadic ICHD probands and blood samples of their healthy parents, and comprehensively analyzed the potential molecular basis of the ICHD phenotypes.

Previous studies evaluating the contribution of somatic variations to CHD pathogenesis applied targeted sequencing of just one or a few known cardiogenic transcription factors on formalin-fixed or freshly frozen tissues. In this study, we performed an exome-wide search for genetic variations in freshly frozen cardiac tissue. The sequencing of DNA extracted from cardiac tissue has the advantage of allowing the identification of tissue-specific post-zygotic variations not being detectable in blood [20]. The contribution of somatic variants identified by ES on cardiac tissue DNA was recently estimated to be around 5% [20]. In this study, we identified six potential somatic exonic variants in cardiac tissue DNA, but only two variants were confirmed as true mosaic variants using the Miseq platform. They both occurred in a gene expressed in the developing heart, but only the *ABCC2* variant had pathogenic in silico predications. Based on current knowledge, we could not establish any CHD-related function for *ABCC2*. Even if future studies would reveal a function of ABCC2 in cardiac development or CHD, the contribution of somatic variants to the ICHD phenotype in our cohort will still have a maximum of 1.4%. Of note, low levels of mosaicism might have escaped the detection threshold resulting from the rather limited standard sequencing depth in ES. The cardiac tissues obtained for DNA extraction were restricted to discarded tissue samples, mostly from the right atrium, and might thus not represent the exact genomic DNA sequence present in the affected tissue.

In the trio-analysis, we focused on rare variants (defined as variants with a gnomAD AF ≤ 0.1%) that were more likely to exert an intermediate or large effect on the phenotype. In total, our cohort contained 20 rare de novo putative protein-damaging variants (one LOF variant and 19 missense variants with pathogenic predictions) in genes being expressed in the developing human heart. Six of these variants (all missense variants) occurred in a gene present in one of the CHD-related panels and resided in six different ICHD patients. This rather low contribution of the de novo variant to CHD-associated genes is in line with the findings of Homsy et al., who found that de novo protein-damaging variants are accountable for only 2% of ICHD patients [44]. In contrast, a strong association has been shown with the inherited variant [45]. We retained ten rare inherited LOF variants and 615 rare inherited missense variants after filtering for expression in developing cardiac tissue, gnomAD AF, in silico predications, and inclusion of the gene in one of the studied CHD-related gene panels. Supplementary Table S5 gives an overview of the 631 variants of interest per ICHD proband, which ranged from 2 to 18 per patient (median 8, mean 8.64, and standard deviation 3.03). Subtle subclinical phenotypes (e.g., small atrial septal defects, patent foramen ovale, and bicuspid aortic valves) and septal defects that closed spontaneously were not systematically excluded in the parents. However, this does not preclude a potential association of the inherited variant to ICHD, especially in view of the incomplete penetrance or multifactorial disease mechanisms. Within the scope of this pilot study, it remains challenging to determine to what extent these variants contribute to the observed phenotypes. The number and effect size of mutated loci needed to reach the disease threshold might be variable within the same disorder [6]. Although these variants may be relatively important, additional factors contributing to the phenotype are likely. In vitro and in vivo disease modeling will hopefully help to further unravel these issues.

We further performed association analyses on the ICHD cohort as a whole, aiming to find associations between ICHD and variants in single genes expressed in developing heart and gene panels of CHD-relevant genes. Therefore, we only focused on rare variants to increase the chances for positive associations.

The analyses showed a statistically significant result for the AT of missense variants in the cilium gene panel, with the ICHD cases containing fewer missense variants than the controls. Homozygous and compound heterozygous pathogenic variants in cilia genes are a known cause of laterality defects e.g., heterotaxy syndromes, atrioventricular septal defects, and outflow tract defects [46,47,48,49]. If confirmed in larger cohorts, this might suggest a protective effect of monoallelic variants.

Though our study is likely underpowered, the TDT does provide an alternative methodological way to identify associations from trio-data.

Overall, we performed an in-depth analysis of coding variation to ICHD, as a sole causal factor or in an oligogenic or polygenic model. This study does not consider the potential contribution of non-coding and structural variations. Small and/or inherited CNVs may contribute within a multifactorial model. Therefore, future studies aiming to unravel the complex multifactorial etiologies of ICHD should be extended to third-generation genome sequencing and other omic technologies to include explorations of non-coding disease mechanisms and epigenetic factors in CHD [6,50].

In conclusion, this study indicated that somatic variants are not a common single cause of CHD. Trio-analysis identified de novo and inherited protein-damaging variants that may contribute to the ICHD phenotype, possibly within an oligogenic or polygenic disease model. Furthermore, our pilot study on TDT and AT provides a framework for the study of larger cohorts to unravel the complex multifactorial etiology of ICHD. Nevertheless, the low uptake rates for explanatory coding variation in ICHD limit the potential of ES in a diagnostic routine.

## Data Availability

The data presented in this study are available in the manuscript or in the Supplementary Materials, or can be obtained from the authors upon written request to the corresponding author.

## Supplementary Materials

The following supporting information can be downloaded at: https://www.mdpi.com/article/10.3390/genes13071214/s1. Supplementary Table S1: Gene panels; Supplementary Table S2: Heart defects in the probands; Supplementary Table S3: Mosaic variants; Supplementary Table S4: High-quality de novo and rare inherited variants; Supplementary Table S5: Variants of interest per proband.
